# Risks and benefits of engaging youth living with HIV in research: perspectives from Kenyan Youth, caregivers, and subject matter experts

**DOI:** 10.1186/s12910-025-01225-1

**Published:** 2025-05-16

**Authors:** Emma Gillette, Winstone Nyandiko, Ashley Chory, Michael Scanlon, Josephine Aluoch, Hillary Koros, Celestine Ashimosi, Whitney Biegon, Dennis Munyoro, Janet Lidweye, Jack Nyagaya, Allison DeLong, Rami Kantor, Rachel Vreeman, Violet Naanyu

**Affiliations:** 1 Department of Global Health, Icahn School of Medicine at Mount Sinai, New York, NY USA; 2 College of Health Sciences, Moi University, Eldoret, Kenya; 3 Academic Model Providing Access to Healthcare (AMPATH), Eldoret, Kenya; 4 Indiana University Center for Global Health, Indianapolis, IN USA; 5 Alpert Medical School at Brown University, Providence, RI USA; 6 School of Arts and Social Sciences, Moi University, Eldoret, Kenya

**Keywords:** Bioethics, Youth living with HIV, Kenya, Research participation

## Abstract

**Background:**

Involving children and adolescents (youth) living with HIV (YLWH) in research is critical for developing appropriate HIV care services and interventions. However, this vulnerable population may not adequately weigh risks against benefits when participating in research, forming an ethical concern, yet little is known about how YLWH perceive these risks and benefits. To inform research-related policies and procedures, we sought perspectives of Kenyan YLWH, their caregivers and subject matter experts (SMEs) on risks and benefits of participation in research in a setting with a high burden of youth HIV infection.

**Methods:**

We conducted a qualitative inquiry on identifying, enrolling, and protecting YLWH (age 10–24 years) in research using semi-structured interviews with YLWH involved in research, their caregivers, YLWH with no prior research experience, and other SMEs at the AMPATH care and research sites in western Kenya. Transcripts were thematically analyzed and emerging themes derived to characterize perspectives of each group on risks and benefits of engaging YLWH in research.

**Results:**

Interviews were conducted with 40 YLWH (50% female; median age 17.5 years), 20 caregivers (70% female), and 39 SMEs [healthcare providers (*N* = 10), community leaders (*N* = 10) community advisory board members (*N* = 4), IRB experts (*N* = 5), clinical researchers (*N* = 6), social science researchers (*N* = 4) and laboratory experts (*N* = 1).] Participants in all groups identified accidental disclosure of HIV status, stigma and discrimination, risks of blood draws, mental health effects, and coercion due to study compensation as risks of research involvement. Benefits fell into 5 categories: clinical, informational, personal, future and community or household benefits. Benefits included access to health care, learning about HIV, gaining hope and community, improving HIV care, and reducing stigma. All participant groups largely held similar views; however, caregivers were the only group to identify misuse of study compensation as a risk, and YLWH less frequently cited clinical benefits.

**Conclusion:**

These findings suggest that participants commonly cite indirect risks and benefits of research participation, yet these are often excluded from institutional guidelines for consent documentation. Researchers should consider including indirect risks and benefits, such as the risk of stigma or the benefit of gaining knowledge and community, to study documentation.

**Supplementary Information:**

The online version contains supplementary material available at 10.1186/s12910-025-01225-1.

## Background

Guidelines for the ethical conduct of research are commonly dictated by the principles of beneficence, no maleficence, autonomy and justice; with an aim to benefit the field of study and participants while minimizing the risk of harm [1]. Ethical research is informed and regulated by offices for human research protections, whose guidelines include particular stipulations for including children and adolescents (youth) as subjects, considering this population as ethically complex and requiring additional protections [2, 3]. Engaging youth living with HIV (YLWH), in particular, requires additional considerations related to their needs during a life stage that involves transitioning from pediatric to adult care, gaining agency over their HIV care management, and navigating disclosure of their HIV status to their community, including family, friends and peers [4–6]. These challenges are further compounded by the risks of HIV-related stigma and discrimination, which influence key components of disease management and therefore long term clinical outcomes, including medication taking and engagement in care [7–11]. Research involving YLWH is critical to informing youth-specific programs and interventions that address their unique needs, particularly because YLWH ages 15–24 worldwide represent 30% of new HIV infections and have high rates of HIV-related morbidity and mortality [12–17]. Part of ensuring such research is ethical includes ensuring participants understand the research protocols in full, including an assessment of the risks and benefits of participation, in order to prevent participant coercion.

Previous studies suggest that youth research participants may not adequately weigh the risks of participation against potential benefits, in part due to limits in decision-making capacity associated with their unique stage of neurodevelopment [18–20]. Adequate consideration of risks and benefits to research participation may be even more important when engaging YLWH, as this population experiences high rates of orphanhood and poverty, and may be at increased risk of coercion when direct benefits such as financial reimbursements are offered [21–23]. Most studies require both assent from the youth participants and consent from the caregiver in order to protect participants under 18 years of age from possible coercion or participation in research they do not understand. Additionally, it is important to understand how YLWH perceive research participation in order to inform the way in which risks and benefits are described in consent and assent forms.

Participant perspectives on research risks and benefits must be assessed in diverse settings, in order to guide enrollment processes, assess protocol comprehension and the use of various incentive strategies. Institutional Review Board (IRB) literature, such as template consent forms, exist to guide the descriptions of relevant risks and benefits in study documentation; however, it is largely left up to the investigators to determine what these are and how to describe them. Current templates suggest a number of potential risks to cite in consent forms, most of which focus on the physical and biological risks of research, rather than social risks such as stigma, discrimination or coercion [2]. Limited literature describes participants’ perspectives of research risks and benefits. Existing studies have suggested that participants report a range of research benefits, from medical services to existential benefits such as the development of life skills [18, 21]. Additionally, studies report that youth participants do not reflect an adequate understanding of research risks and that some forget the risks because they are focused on the benefits offered [18, 21].

Despite commonly citing that youth may not accurately weigh research-related risks and benefits, there is limited evidence on what YLWH and other subject matter experts (SME) actually perceive as risks and benefits of participation in HIV research. While studies have been done with adolescents at high risk of contracting HIV, no studies have sought the perspectives of YLWH, nor their caregivers. These perspectives are critical, particularly among YLWH who are perinatally-infected, and have been participating in HIV research for several years. Moreover, the majority of perinatally HIV-infected YLWH live in settings where HIV-related stigma is both pervasive and dangerous. These topics are necessary to informing institutional, national and international policy regulating research and ethics procedures. This study aims to address this gap in literature by exploring the perceptions of Kenyan YLWH, their caregivers, and other SMEs on the risks and benefits of youth participation in HIV research; these findings may therefore inform research-related policies and procedures.

## Methods

### Study setting

This study was conducted at the Academic Model Providing Access to Healthcare (AMPATH), a clinical and research collaboration between Moi University School of Medicine, Moi Teaching and Referral Hospital in Eldoret, Kenya and a consortium of North American universities [24, 25]. AMPATH provides care for more than 7,000 children and YLWH, and is also the home of the Rafiki Center of Excellence in Adolescent Health, a large adolescent health center.

This study was conducted within a broader, ongoing clinical cohort study that was originally assembled in 2010–2013, enrolled participants in two completed longitudinal studies, and is currently re-enrolling in a third study focused on adherence and drug resistance among YLWH (NIH Grants 1R01 AI147333, 5R01 AI120792, and 1 K23MH087225). This series of studies longitudinally follows YLWH as they grow into adolescence in western Kenya, with an aim to assess the associations between antiretroviral therapy (ART) adherence, treatment failure and the development of drug resistant mutations. The studies collect blood samples for viral load and drug resistance testing, which informed some of the interview questions for the present qualitative study. Eligibility for the original parent study was: (1) perinatally infected with HIV, (2) ≤ 14 years of age at enrollment, (3) on or initiating 1 st-line Non-Nucleoside Reverse Transcriptase (NNRTI)-based ART regimens, and (4) receiving HIV care at an AMPATH clinic. Participants were initially approached during routine clinic visits or via phone call, and no participants refused participation.

### Study design

To gain a comprehensive range of perspectives, this study enrolled four categories of participants: (1) YLWH who are currently enrolled in the parent clinical cohort study, (2) caregivers of YLWH enrolled in the parent study, (3) YLWH who have not previously participated in research, and (4) SMEs. The results present YLWH with and without research experience together, as perspectives were consistent across those groups.

The interviews focused on the identification, enrollment and protection of YLWH in longitudinal clinical research, and probed issues related to age of consent and assent; how research involvement changes from childhood through adolescence and into adulthood; involvement of caregivers in research; protections against accidental disclosure of HIV status; and considerations related to research with a population with low socioeconomic status, high orphanhood, and living with HIV. This paper discusses the risks and benefits cited of youth research participation as identified by study participants.

### Sampling and recruitment

YLWH were recruited from our study cohort based on previously scheduled study visits for the parent study (for those enrolled in the parent study) or previously scheduled clinic visits at the Rafiki Center of Excellence in Adolescent Health (for those not previously engaged in research). Youth participants were included only if they were (1) between 10 and 24 years of age and (2) aware of their HIV status. Caregivers were similarly recruited through random sampling from scheduled visits. Caregiver participants were eligible if they were (1) a caregiver of a YLWH enrolled in the parent study, (2) 18 years of age or older, (3) aware of the HIV status of the youth participant, and (4) knowledgeable about the care and research participation of the youth participant. Subject matter experts (SMEs) were identified by the research team as experts in community leadership, healthcare provision, research, ethics and policy. SMEs were eligible if they were: (1) 18 years of age or over and (2) determined by the study team to be a SME in one of the following groups: community leaders (village elders and chiefs), members of adolescent or community advisory boards, healthcare providers, members of international and local IRBs, clinical or social science researchers from the AMPATH Research Network and other institutions conducting research with YLWH in Kenya and research laboratory leadership. Interviews were conducted from March to May 2021 and participants were recruited until saturation was reached.

### Data collection and analysis

Two Kenyan facilitators experienced in qualitative interviewing and trained on the study protocol (one male, one female) conducted interviews in either Kiswahili or English, depending on the participant’s preference. Facilitators had no prior relationships with participants, and no other observers were present for interviews. Interviews with youth, caregivers and most SMEs were conducted in private rooms in the clinic setting. One village Chief was interviewed in his private office, and IRB members were interviewed virtually via Zoom. Interview guides were tailored to probe topics relevant to each participant group. Interviews were audio-recorded, transcribed, and those conducted in Kiswahili were translated into English for analysis; no data or notes were captured in the field. Deductive thematic analysis was led by two researchers (HK and EG) based on an initial coding framework derived from the interview guide and reviewed with JA and AC. The researchers independently extracted data using the qualitative software program NVivo, version 12 [26]. The same investigators (HK and EG) led inductive analysis, and extracted emerging themes independently before comparing results and reaching consensus on relevant themes. Data collection and analyses were conducted in line with the Equator Network Standards for Reporting Qualitative Research (SRQR).

## Results

Ninety-nine interviews were conducted with 40 YLWH (20 involved in research—50% female, median age 17.5 years; 20 not involved in research—50% female, median age 18 years), 20 caregivers (70% female), and 39 SMEs (44% female). SME participants included healthcare providers (*N* = 10), community leaders (*N* = 10) community advisory board members (*N* = 4), IRB experts (*N* = 5), clinical researchers (*N* = 6), social science researchers (*N* = 4) and laboratory experts (*N* = 1). Both YLWH who were and were not involved in the parent study often shared similar views. Results are presented in aggregate unless presented otherwise.

### Participant cited risks

Overall, participants primarily identified risks in two categories: risks that were a *direct result* of participation in a study, and risks associated with the *behaviors and social factors* surrounding research participation (Table 1).

**Table 1:** Examples of participant-cited risks of youth research participation

Direct Research Risks	Blood Draw Complications	“I think one of the risks that I even think about myself is when your blood is being sampled and taken for a blood test then all of a sudden the needle breaks inside your arm.” –*adolescent above 18 in R01, male* “Yes, infection from, let’s say, the blood sample, maybe the tools that they used for blood sampling can lead to infection. Maybe they were not sterilized well, yes…in which they can lead into infection.” –*adolescent below 18, research-naïve, female*
	Ineffective or Dangerous Experimental Drugs	“The only risk that can be there is if it is a research study that aims to research at a particular drug that hasn’t been proved, there will be an effect because they will stop using the initial drug and switch to the one being researched on, and during this certain period the viral load will go high because the drug has not been approved and it is not working, so by the time they realize this, the viral load will have shoot high…” –*caregiver, male*
	Harassment or Intimidation in Research	“…maybe when they get to the clinic where they are going, and then they are harassed then the child might refuse to come another day… as in being harassed…someone who maybe does not know how to talk in low tones like for example like for my daughter whom I have brought, then someone shouts at her ‘Why are you not talking?’ So you know my child will develop some [fear] so she cannot agree again to come to that clinic.” – *caregiver, female*
	Accidental HIV Disclosure to Child through Research	“As I had said before, some young people do not know why they take drugs, and they may learn why they take drugs in research. You don’t know how it could affect the child. It can lead to something bad like being in denial and in the process, they may stop taking the drugs which will increase the viral load.” –*adolescent below 18, involved in R01, female*
	Accidental HIV Disclosure to Others	“Disclosing the child’s status by accident isn’t a good thing because when a negative person finds out he will go to tell others and when the child passes by they will be gossiping that he has HIV and they should keep away from him. The child will be stigmatized. It will be risky if the information leaks. We don’t know how we can keep the secret but we should just try so that we don’t ruin their lives.” –*caregiver, female*
	Mental Health Risks	“Emotional injury. In the case where the researcher asks intense question to the participant, that may later lead to the participant wanting to maybe commit suicide and can also lead to depression sometimes.” –*adolescent above 18, research-naïve, male*
	Coercion with Financial Compensation	“Again, they may just want to participate in as many research [projects] as possible especially when they know there's compensation, whether or not it affects them. Because they're vulnerable and you know that some of these adolescents are even orphaned, especially for those who were born with HIV, some of their parents have died. And we know some circumstances are very difficult, and so they can view research as a way of getting some money even when their own wellbeing is compromised…” –*clinical researcher, female*
Associated Behavioral/Social Risks	Stigma	“One of them is the most dangerous one, is stigma. You know when they come here, then when they go back home, maybe their peers will ask them ‘where were you, what happened to you?’ Then they might expose whatever they come to say. That stigma will be there. In a community, there are so many creations of stories, creation of rumors or words. It might even lead one to hang himself or herself.” –*chief, male*
	Discrimination	“They will discriminate you because they don’t have any more knowledge about HIV so they just see anyone with HIV even they cannot share cups with you because they just see you can transmit with them. But that’s not true… they may have negative thoughts about you when you don’t have any intentions.” –*adolescent above 18, research-naïve, female*
	Youth Demoralizing Each Other	“The only bad thing that may happen is when they are together participating in the research and they start saying negative things about the research study, that will tend to demoralize them. When they are together without the research team. You may never find out because they will meet in other places and share research experiences. This can demoralize them from participating in the research.” –*caregiver, female*
	Self-Stigma	“Self-stigma, am talking about a situation where the individual feels, feels less confident, feels less worthy, feels less important, you know? Negatively treats themselves, negatively discriminates against themselves on the basis of their status.” –*community leader, female*
	Irresponsible Use of Monetary Compensation	“If you give them something we won’t know because they are adults so the risk that I can see is according to today’s world, there are a lot of things like bhang and the child can buy because he has money.” –*caregiver, female*
	Loss of Confidentiality	“I don’t think that there is anything bad that can happen to you because they are informed; unless if the results are exposed or used for someone’s benefit or gain instead of being kept private.” –*caregiver, female*
	Risk of Being Left Out of Benefits	“There is the risk of being left out of the benefits that come out of that research because benefits come much, much, much later and so…and there adolescents may not…you know adolescents especially the girls maybe married away and so they get left out of some of these as the benefits come to the community where the research was taking place and once you marry away now you lose the benefit, some of the things that I see.” –*clinical researcher, male*
	Caregivers Taking Financial Compensation	“…young people shouldn’t be involved in a research… in this research, maybe there is some sort of appreciation [compensation… Because maybe this caregiver is the one who takes the appreciation [compensation] and not the child. But after the child notices that, when the research dates reach, maybe this child will no longer wish to participate in the research because the caregiver will be given the incentive. The child won’t benefit.” –*adolescent above 18, involved in R01, male*
No Risks		“It is good if they participate, I don’t see anything bad happening.” –*caregiver, female* “I don't think there are any risks.” –*adolescent below 18, involved in R01, male*

### Direct risks of research involvement

All groups of participants identified both physical and mental risks of research participation, including infection following blood draws and emotional injury following intense questions. Accidental disclosure of HIV status, both to the YLWH themselves and to others, were concerns, particularly among caregivers and YLWH participants. Some YLWH also explained that the fear of accidental disclosure to others may prevent them from participating in research.

Some caregivers and SMEs discussed the risk of being given experimental treatments that have no effect or cause adverse side effects or outcomes. A minority of participants, primarily caregivers, cited harassment or intimidation by researchers as a risk of participation. SME participants expressed concern about YLWH being coerced to participate in research when monetary compensation is offered, citing that younger participants may be orphans, may not have money of their own or may have access to fewer financial resources.

### Behavioral and social risks of research involvement

All groups of participants identified experiences of stigma and discrimination, irresponsible use of study compensation, and being left out of benefits such as receiving research results or monetary compensation as risks of research participation. Social risks were a major concern. YLWH, caregivers and SMEs most commonly cited the risk of stigma and discrimination following a loss of confidentiality in a study, and highlighted the connection between experienced stigma and mental health symptomology, suicidal ideation, and self-stigma. Participants also expressed concern that youth participants may discourage each other from participating in research. Notably, many YLWH participants cited accidental disclosure and associated stigma and discrimination not only as risks of participation, but as reasons to avoid participation all together. All participant groups detailed different possible risks associated with receiving monetary compensation for participation, including caregivers thinking that YLWH will spend money on cannabis (referred to as *bhang* in Kenya) or alcohol without their knowledge. Some caregivers suggested that researchers should have a role in ensuring the compensation is used properly. Some YLWH participants also cited that there was a risk caregivers would receive their compensation instead of the participants, particularly because caregivers may exploit youth participation for this compensation.

A minority of participants expressed that there were no risks associated with participating in research. Some SMEs and caregivers described that, even if confidentiality was lost, there is no longer a risk of stigma or discrimination in their communities, following increased education and sensitization.

### Participant-cited benefits

Participants identified benefits broadly falling into five categories: clinical, informational, personal, altruistic and community or household benefits (Table 2). All participant categories identified improvements in medication adherence as a benefit, with some YLWH further suggesting that their improved adherence encouraged their peers to improve their adherence as well. Participants cited improved clinic attendance and a higher quality of clinical care, including improved access to medication, as benefits of research participation. Most participants highlighted that participation in research led to higher quality and lower cost clinical care, which may not normally be accessible. Participants commonly cited opportunities to continue to learn more about HIV and care management, informational benefits that YLWH reported sharing with their peers. Learning about study results was considered a benefit by some participants, which would motivate them to participate in research again in the future.

**Table 2:** Examples of participant-cited benefits of youth research participation

Clinical Benefits	Improved Medication Adherence	“They will feel like their good drug adherence is the reason they are participating in research, and this will encourage them to continue to use drugs…. Another benefit is their friends who are not part of the study will be encouraged to also improve on their adherence too, to be like the friend.” –*adolescent above 18, involved in R01, female*
	Improved Clinic Attendance	“I think they also improve their adherences moving forward, both medications, and both visits, because of a personalized kind of interaction, they also get to learn a lot.” –*health care provider, male*
	Higher Quality of Clinical Care	“To themselves I think there will be better quest to get more knowledge, sometimes to get direct care, sometimes they get trial drugs which are superior, sometimes they get fancy things that can help them to remind them about drug adherence, so there is an improved quality of care mainly to the patients who are in research, better outcomes-viral suppression and retention because they are seen more closely and followed up with phone calls and many things making the quality of care better.” –*health care provider, male*
	Access to Medication	“The benefit I would seek is that sometimes some clinical research has benefit in itself in that the participants are able to access medication they would never access if they were not participating in the research.” –*community leader, female*
Information Benefits	Learning of Study results	“The other opinion that I have is that once a research study has been successful, those involved should also benefit because they can have seminars to share the information received with others so that they will motivated to participate in a research.” –*caregiver, male*
	Gaining Knowledge about HIV	“Another deeper benefit which we rarely think about is knowledge. In the process to participating in research, people actually gather a lot of information that they would not otherwise access. So they may get to know quite a bit and sometimes this knowledge is helpful for them in management of their conditions particularly when you are dealing with regular diseases like HIV.” –*community leader, female*
	Sharing Information with Others	“They can share the same information with their friends who are also living with HIV. They can also become doctors when they grow up and share the information with other patients.” –*adolescent below 18, research-naïve, male*
Personal benefits	Acceptance of Status	“It also helps someone to accept themselves. Because living with HIV is not the end of living. As long as you take your drugs and exercise it well. There is still a future for you, and you can still make it.” –*adolescent below 18, involved in R01, female*
	Financial Compensation	“Another thing is that by involving them in research, it may enable them also to get some stipend or reimbursement that will assist them obtain their basic needs.” –*chief, male*
	Getting Advice	“And then, you know when we leave them there with the doctor, I see it is good advice that she is given she is told to read hard be determined, take your medication well, and I see that too to be of benefit.” –*caregiver, female* “They get advice from the doctors, they are told to eat balanced diets, have regular exercises, they should try and live stress-free lives which is a bit difficult. When they follow what they are being told you find that most of them can live a long life.” –*chief, male*
	Having Community and Social Support	“The benefits I get are like the encouragement I get to continue moving on so that the more I continue taking the medication which also helps to know my status and how I am, it also helps to abstain from the discrimination from others.” –*adolescent above 18, involved in R01, female* “Apart from gaining knowledge, yeah, they also get a chance to associate themselves with people who have lived longer with HIV and who are living healthy with HIV so they tend to learn the skills from them. They also learn on how to cope up with it.” –*adolescent below 18, research-naïve, female* “The research sometimes it helps us know the background of these children…youths, sometimes they open up to the researchers more because they know they are going to help them in knowing more of about their condition, so of the time when we link up together we get to know so many things that these youths are going through.” –*health care provider, male*
	Improved School Attendance	“For example, schooling, they won’t drop out of school.” –*caregiver, female*
	Opportunity to Share Experiences	“Personally I think it’s a nice feeling when you participate in research [projects], when you talk with people maybe that person had something to air and never knew where to take it, but when research comes ensure that that person gets the opportunity to say what he/she thinks which is a good feeling because when you say something and someone hears you it’s a very nice feeling.” –*community advisory board member, female*
Altruistic Benefits	Development of HIV Care and Policy	“The benefit might not be direct to the one participating, but the data which will be collected will help in the future management of these adolescents. It might not be direct to the person you are dealing with at that moment. It might help in the long run of managing of adolescents and also help the health care workers in managing.” –*health care provider, male* “There is also benefit because not to this participant, but also to the research community. Yes because at least information is received that informs policy and improves care for generation and generations to come.” –*laboratory lead, female*
	Improvements to Youth-Friendly Care and Interventions	“From the intervention and then at the policy level policy making, and even the new drugs, new whatever, new devices, new inventions that are focusing on adolescence will be able to assist this age group to be HIV protected or live with HIV in a better way.” –*clinical researcher, male*
Community or Household Benefits	Reduces Stigma	“It reduces stigmatization. They can also educate others who don’t know about their status.” –*caregiver, female* “Like the research I was previously involved in, it makes someone feel more confident and you learn not to stigmatize yourself. It helps you grow.” –*adolescent above 18, research-naïve, female*
	Household Benefits	“There also some research that not only take care of the participant, but also the household…So you find the household also gets to benefit from this research. Because for example, if there are studies that give medication, you also ensure that you also get good diet, because I mean, how do you take medicine without diet? …. Or if there are studies that take care … of a participant and they find that the cause of maybe HIV, there could be other effects in the household. You find that some studies take care of such… So the household gets to benefit. And again, by participating in the research, the quality of life is improved. So it is less burden into the household… they do not spend a lot of money in… hospital visits. There is happiness because at least somebody is never sick forever or always.” –*laboratory lead, female*
No Benefits		“There are no benefits that they get.” –*adolescent above 18, involved in R01, female*

All categories of participants cited personal benefits, ranging from being given hope and advice to receiving financial compensation. YLWH participants commonly cited benefits to their personal development, such as learning to accept their HIV status, receipt of encouragement and support, and being given the opportunity to express themselves and share their experiences. Some caregiver and SME participants described YLWH receiving advice, developing relationships with researchers, finding community and improving their school attendance as major benefits. A number of SMEs and YLWH participants cited financial compensation as a benefit, with some YLWH citing they thought that compensation included jewelry or food.

Many participants, in all categories, cited future benefits, particularly contributing to developments in HIV care and policy. Multiple participants mentioned feeling that they were helping work towards a cure for HIV and informing policies for practice, and that it was beneficial for them to contribute to the improvement of care “*for generations and generations to come*”. SME participants in particular discussed the benefit of the research contributing to the development of adolescent-specific care specifically, and emphasized that learning about and addressing issues specific to YLWH are major benefits of participation. A number of participants cited community and household benefits, including offering compensation to ease their family burden to pay for hospital visits, medication and maintenance of a good diet. Some caregivers and YLWH mentioned that research participation is beneficial to reducing stigma, as it helps limit self-stigmatization and sensitizes others to the condition. A minority of participants expressed that there were no benefits involved with research participation.

### Weighing of risks and benefits

Interviews did not specifically probe participants’ views on how to weigh research risks and benefits; however, some participants expressed that YLWH may not consider all of these aspects when deciding to participate in research, and that they may place greater weight on benefits than risks.

*‘A young person will not consider all those issues, there is a person who will just think oh, this is a research [study], how much am I getting you know, they tend to focus more on the benefits than on the risks, young people are risk takers generally so they might not necessarily conceptualize the level of risk they are involving themselves in to.’ –social science researcher, female*

Other SME participants expressed that HIV-related stigma is associated with both risks and benefits of research. Participants described that youth may experience stigma or discrimination as a result of research participation; at the same time, research works to reduce stigmatizing attitudes and behaviors by educating YLWH and others about HIV.

*‘Sometimes I think it can be both negative—because they might get discriminated [against] but on the other side again, it can even help other people who were already fighting stigma and discrimination to actually realize that, so I am not alone, there are other people who are also living positively. It’s two-way.’ –health care provider, female*

## Discussion

This study sought to present the perspectives of YLWH, their caregivers, and other SMEs on the perceived risks and benefits of youth participation in HIV-related research. Ninety-nine participants provided versatile perspectives on the risks and benefits of engaging YLWH in research, with largely similar views across research-experienced YLWH, research-naïve YLWH, caregiver and SME participant groups. Both groups of YLWH participants expressed similar sentiments about research participation, possibly due to the shared experiences of YLWH in both groups in the community fostered at the clinic where participants receive HIV care.

Participant groups differed in perspectives on study compensation and identification of benefits, with more caregivers reporting concerns about irresponsible use of study compensation, and YLWH tending to identify personal, altruistic and informational benefits more than clinical benefits. This difference between caregiver and YLWH perspectives could be relevant for the development of consent and assent documentation, as caregivers may not be aware of the importance of indirect benefits to YLWH, and should consider these when deciding whether their child participates in research.

Participants identified a number of clinical risks, including the risks associated with blood draws and the mental health impacts associated with difficult questions, such as questions probing suicidal ideation or experiences with sexual assault. Accidental disclosure, stigma and discrimination were the most common risks, identified by all groups, associated with engaging YLWH in research. The impact of HIV-related stigma on people living with HIV is well understood, with pervasive effects influencing medication taking behaviors, engagement and retention in clinical care, viral suppression, and the development of community support, among others [27–32]. Despite common concerns about stigma and discrimination as risks of research, IRB documentation such as consent templates and guidelines often overlook these risks, primarily citing physical risks associated with blood draws and other research activities. Previous studies have investigated perceived risks of engagement in research, but from the perspective of youth at risk for HIV [18, 21]. Participants in these previous studies identified similar risks as were expressed in this study: pain from a needle prick, discomfort surrounding difficult questions, and possible coercion of youth when compensation is offered [18, 21]. Some participants in our study suggested that participation in research involved no risk, citing that they trusted researchers to maintain confidentiality and that stigma and discrimination were no longer issues in their community.

Participants cited a range of benefits related to clinical, personal, informational, future HIV care and household or community perks of involvement in research. These findings are largely consistent with previous work, which described benefits as informational, existential, emotional, medical and material [18, 21]. Participants commonly cited access to clinical care as a benefit, particularly for its high quality and low cost. Although many participants feel that a higher level of care is a benefit, IRB guidelines stipulate that “enhanced observations,” meaning an increased level of attention in clinical care and more frequent clinical observation, and improved care due to researcher oversight are not to be listed as benefits in study documentation [2, 33]. All categories of participants also cited community and social support as a benefit of participation; studies have illustrated that for YLWH, social support is protective against some of the harmful consequences of HIV-related stigma, and can lead to improved clinical outcomes [7, 34–40]. These findings suggest that participants are correct in their perceptions of the benefits of research, and that involvement in research is an important facilitator of social support for YLWH.

This study has a number of strengths. We enrolled participants of varying backgrounds to provide a wide breadth of perspectives on the topic, including YLWH who have been involved in research for years, YLWH who are not involved in research, caregivers of YLWH involved in research, and experts in a number of different fields. Additionally, two researchers conducted separate coding of the transcript data and had conflicts resolved by an additional two researchers to ensure interrater reliability and minimize bias in the analysis. The analysis is limited by the fact that participants’ responses may have been influenced by social desirability bias, and participants may have been reluctant to share negative opinions about research out of fear of being excluded from future research and associated benefits. Additionally, the study did not involve caregivers of YLWH who did not participate in research, who may have expressed opinions which are not presently represented. This study was conducted at a well-established clinic providing care for YLWH in western Kenya, which may not be generalizable to other settings. The analysis of the data may have been limited by the researchers’ personal reflexivity and cultural backgrounds; however, we attempted to mitigate this by having both a Kenyan and American analyst independently analyze the data and confirm the themes, and scientific oversight was provided by a team of both Kenyan and American investigators. This study is also furthering prior analyses conducted of these interviews, which focused on the ethical considerations surrounding the involvement of YLWH, by delving deeper into the specific topic of the risks and benefits of involvement [41]. This information can be used to inform study protocols, ethics committee guidance and information provided to research participants.

## Conclusion

YLWH, their caregivers, and SMEs recognized the importance and need for YLWH to participate in research, noting an array of both benefits and risks of participation. Our findings suggest that consent and assent documentation may not adequately inform participants on study related risks or benefits. Researchers and IRBs should consider revisions to the standard consent and assent templates to include participant-identified psychosocial and social risks. These findings may also inform research study designs in identifying appropriate benefits to offer participants, including compensation and results returned to research participants. Future directions for research may include identifying methods of mitigating the risks of research identified by YLWH, their caregivers, and SMEs, and ensuring that research participants receive the benefits identified in these analyses.

## Supplementary Information

Supplementary Material 1.

Supplementary Material 2.

## Data Availability

The data will be made available upon request to the corresponding author.
