# Supplementary material for: Risks and benefits of engaging youth living with HIV in research: perspectives from Kenyan Youth, caregivers, and subject matter experts
Source: BMC Med Ethics. 2025 May 16;26:63. doi: 10.1186/s12910-025-01225-1 (PMC12083134; doi:10.1186/s12910-025-01225-1)
Supplement: Supplementary file 1 — Supplementary Material 1. [file 12910_2025_1225_MOESM1_ESM.docx]

**Study Title:** Addressing bioethical research gaps in research with YPLWH in Kenya

#### **Principal Investigators (Head Researchers):** Dr. Rami Kantor, MD, Dr. Rachel Vreeman, MD, MS, and Prof. Winstone Nyandiko, MBChB, MMED, MPH

**Interview Guide for YPLWH and Caregivers**

*[PRIOR TO RECORDING, INSTRUCT PARTICIPANTS THAT THEY SHOULD NOT SAY THEIR NAME OR ANY OTHER PRIVATE INFORMATION THROUGHOUT THE INTERVIEW].*

*General perspectives on research with YPLWH*

1. First we would like to ask you some general questions about research.

-What does the idea of research mean to you?

-What do you think are important issues for a youth living with HIV to consider when they make decisions about a research study?

-What are important issues for their family to consider?

-What would you want the researcher or the clinic to understand about what should be done when they identify youth to be involved in research?

-How do these issues impact younger YPLWH (under 18 years of age) versus older YPLWH (over 18 years of age)?

*[FOR YPLWH WHO HAVE NOT PREVIOUSLY PARTICIPATED IN RESEARCH, ASK QUESTION 2. FOR YPLWH AND CAREGIVERS INVOLVED WITH THE PARENT R01, SKIP TO QUESTION 3]*

2. Have you heard any stories about YPLWH or other young people who participate in research studies? What have you heard?

3. Sometimes, when researchers want to enroll YPLWH in research, they review the medical records and files from a clinic in order to find YPLWH.

-What do you think about that approach?

-Do you think it is okay? (Why or why not?)

-What would you suggest researchers do to find the right youth for research studies?

-How would you want the researchers to find youth for a study?

4. How and where do you think researchers should approach YPLWH for potential involvement in research? (E.g., in a clinic setting during routine care, in a community setting like a school, at your house through a community health or outreach worker, etc.)

*[FOR YPLWH AND CAREGIVERS INVOLVED IN THE PARENT R01]*

4a. How were you recruited for research studies in the past? Describe what this process looked like for you and your family. Would you change anything about how the research team approached you?

5. When you think about YPLWH who are younger, especially those under 18 years of age who are still considered minors or children who need permission from caregivers to do things like participate in research:

-How should parents or caregivers be involved in research with YPLWH?

-Should caregivers be required to provide their consent for YPLWH to participate in research?

-What about for YPLWH who are over 18 years of age?

-How does the process change as a child gets older?

-Are there any cases where the caregivers should not be involved or reasons why it may be harmful to the YPLWH to have their caregivers involved?

-Are there cases when caregivers could exploit children during research, meaning that they enroll them for their own benefit and not for the benefit of their child?

6. What are some of the risks or bad things that could happen when YPLWH participate in clinical research? In other words, do you think there are any negative consequences or bad things that could happen to YPLWH by participating in research?

Probe: Accidental issues of disclosure of HIV status, HIV-related stigma and discrimination

7. Do you think there are certain things that make YPLWH more vulnerable to risks or potential negative consequences compared to other groups of potential research participants? Are there things researchers should do to specifically protect YPLWH in research?

8. What are some of the benefits or good things that happen when YPLWH participate in clinical research? In other words, do you think there are positive things that could happen to YPLWH by participating in research?

9. Some research involves collecting and analyzing participants’ blood.

-Do you have any concerns about research that collects and analyzes blood from participants?

-What are these concerns?

-What about for research with YPLWH, are there specific concerns or recommendations?

10.When researchers do collect blood specimens for testing and analysis for research, do you think that researchers should be responsible for sharing the results of any tests with research participants and/or their caregivers?

11. Some research involves storing participants’ blood, either to use later in the research study or to keep in a research laboratory and potentially use for research at a later time.

-Do you have any concerns about research that collects and then stores blood from participants?

-What are these concerns?

-What about for research with YPLWH, are there specific concerns or recommendations?

*[FOR YPLWH AND CAREGIVERS INVOLVED IN THE PARENT R01]*

Probes: Own experience with having blood stored and whether/why they consented or not

12. Sometimes, after a researcher has stored blood from participants for one research project, they want to do new tests on that participant’s blood for reasons that are different from the original research study.

-Do you think researchers should be required to contact that participant and get permission to do additional tests on their blood?

-Do you have concerns about this, and why?

-Do you think there are specific concerns or reasons as to why this might be different for research with YPLWH?

-What would you recommend that researchers do when they want to do new tests on blood that is stored?

13. Sometimes, after a researcher has stored blood from participants for a research project, they want to share blood samples with other researchers or organizations like governments who are interested in building up large databases of samples.

-Do you think researchers should be required to contact that participant and get permission to share their blood samples with other researchers or organizations?

-Do you have concerns about this and why?

-Do you think there are specific concerns or reasons as to why this might be different for research with YPLWH?

Probe: Sharing HIV resistance data in specific, sharing genomic data in specific.

14. In research that does store participants’ blood in a research lab, there are different ways to identify that blood sample. For example, sometimes that blood sample can be stored with participants’ name and other identifiable information, or it can be stored using a random study number so that we do not know the name of the person that the sample comes from.

-What do you think are the benefits and risks of storing participants specimens in these different ways?

-Would you recommend that researchers stored them in a specific way?

-What about for research with YPLWH, are there specific concerns or recommendations?

*[[Questions specific to the procedures and activities of the parent R01 and other studies. FOR YPLWH AND CAREGIVERS INVOLVED IN THE PARENT R01 ONLY]]*

15. As you know, we have been following you/your child in several different research projects related to adherence to medicines and drug resistance.

-In general, how have you felt about participating in these research projects?

-What have you liked about participating in these research projects?

-What have you disliked about participating in these research projects?

Probe this question in the context of (and specific to YPLWH):

- Identification and first contact with study participants
- Consent / assent processes for enrollment
- Data collection and follow up
- Benefits and risks to participation
- Adequacy of protections against risks

In the current research study, we are collecting blood from you/your child to test your viral load and any potential drug resistance. During the consent process, we asked you/your child different questions about if and how we could use and store your/your child’s blood sample. We would like to know more information about how you made these decisions about how we can use your/your child’s blood sample.

16a. Did you give us permission to store your information and blood specimen to use in future research studies? What does that mean to you? What made you decide to allow/not allow use to use your information and blood specimen in future research?

16b. If you did allow us to keep your blood specimen for future research, we asked you how you would like that blood specimen stored in our research laboratory. You/your child could either have your/their specimen stored that in linked to your/their identify (name, age, etc.) or you could have it store anonymously. What does that mean to you? What did you choose and why?

16c. We asked if we have permission to contact you/your child in the future to collect additional information about you/your child, discussion how your/your child’s information and specimens might be used, or to discuss your/your child’s participation in another research project. What did you choose and why? Do you have any concerns with being contacted again? How would you like to be contacted?

16d. We asked if we have permission to keep the information and specimens indefinitely and use them for future studies that are directly related to the purpose of the current study. What did you choose and why? Do you have any concerns about us using your information and specimens in the future?

16e. We asked if we have permission to keep the information and specimens indefinitely and use them for future studies that are not directly related to the purpose of the current study? What did you choose and why? Do you have any concerns about using your information and specimens in the future for different studies that you do not know about?

**(c) Subject matter experts** (community leaders, members of youth and community advisory boards, healthcare providers, members of IRBs, researchers, laboratory heads, and government representatives)

*General perspectives on research with YPLWH*

1. What do you think are some important issues to consider when identifying and enrolling YPLWH in clinical research in this setting? How do these issues impact younger YPLWH (under 18 years of age) versus older YPLWH (over 18 years of age)?

2. If researchers want to enroll YPLWH in research, do you see any potential ethical or other problems with researchers being able to search medical records and clinical databases to find YPLWH that meet the criteria for a research study?

3. How and where do you think researchers should approach YPLWH for potential involvement in research? (E.g., in a clinic setting during routine care, in a community setting like a school, at home through a community health or outreach worker, etc.)

4. Especially for YPLWH who are younger (under 18 years of age), how should caregivers be involved in decisions related to the participation of YPLWH in research? Should caregivers be required to provide their consent for YPLWH to participate? What about for YPLWH who are over 18 years of age?

5. What are some of the risks of YPLWH participating in clinical research? In other words, do you think there are any negative consequences or bad things that could happen to YPLWH by participating in research?

Probe: Accidental issues of disclosure of HIV status, HIV-related stigma and discrimination

6. Do you think there are certain things that make YPLWH more vulnerable to risks or potential negative consequences compared to other groups of potential research participants? Are there things researchers should do to specifically protect YPLWH in research?

7. What are some of the benefits of YPLWH participating in clinical research? In other words, do you think there are positive things that could happen to YPWH by participating in research?

8. Some research involves collecting and analyzing participants’ blood. Do you have any concerns about research that collects and analyzes blood from participants? What are these concerns? What about for research with YPLWH, are there specific concerns or recommendations?

Probe: Responsibilities of researchers to provide feedback/share results of any tests

9. Some research involves the storage of participants blood, either to use later in the research study or to keep in a research laboratory to potentially use for research at a later time. Do you have any concerns about research that collects and then stores blood from participants? What are these concerns? What about for research with YPLWH, are there specific concerns or recommendations?

10. Some research, after they have stored blood from participants for a research project, want to do tests on that participant’s blood for reasons that are different from the original research study. Do you think researchers should be required to contact that participant and get permission to do additional tests on their blood? Do you have concerns about this and why? What about for research with YPLWH, are there specific concerns or recommendations?

11. Some research, after they have stored blood from participants for a research project, want to share blood samples with other researchers or organizations like governments who are interested in building up large databases of samples. Do you think researchers should be required to contact that participant and get permission to share their blood samples with other researchers or organizations? Do you have concerns about this and why? What about for research with YPLWH, are there specific concerns or recommendations?

12. In research that does store participants blood in a research lab, there are different ways to identify that blood sample. For example, sometimes that blood sample can be stored with participants’ name and other identifiable information, or it can be stored using a random study number so that we do not know the name of the person that the sample comes from. What do you think are the benefits and risks of storing participants specimens in these different ways? Would you recommend that researchers stored them in a specific way? What about for research with YPLWH, are there specific concerns or recommendations?

13. Are you involved in research with YPLWH? How so?

14. If you are involved in research with YPLWH, what are some of the ethical challenges you have faced in this research? How have you attempted to handle or address these challenges?

15. What guidelines and policies are in place in your setting to inform ethical research with YPLWH?

16. Are you involved in research with biological sampling and biobanking? How so?

17. If you are involved in research with biological sampling and biobanking, what are some of the ethical challenges you have faced in this research? How have you attempted to handle or address these challenges?

18. What guidelines and policies are in place in your setting to inform ethical research with biological sampling and biobanking?
