# Supplementary material for: Risks and benefits of engaging youth living with HIV in research: perspectives from Kenyan Youth, caregivers, and subject matter experts
Source: BMC Med Ethics. 2025 May 16;26:63. doi: 10.1186/s12910-025-01225-1 (PMC12083134; doi:10.1186/s12910-025-01225-1)
Supplement: Supplementary file 2 — Supplementary Material 2. [file 12910_2025_1225_MOESM2_ESM.docx]

**Study Title:** Addressing bioethical research gaps in research with YPLWH in Kenya

#### **Principal Investigators (Head Researchers):** Dr. Rami Kantor, MD, Dr. Rachel Vreeman, MD, MS, and Prof. Winstone Nyandiko, MBChB, MMED, MPH

**Interview Guide for Subject Matter Experts**

*[PRIOR TO RECORDING, INSTRUCT PARTICIPANTS THAT THEY SHOULD NOT SAY THEIR NAME OR ANY OTHER PRIVATE INFORMATION THROUGHOUT THE INTERVIEW].*

**Subject matter experts** (community leaders, members of youth and community advisory boards, healthcare providers, members of IRBs, researchers, laboratory heads, and government representatives)

*General perspectives on research with YPLWH*

1. What do you think are some important issues to consider when identifying and enrolling YPLWH in clinical research in this setting? How do these issues impact younger YPLWH (under 18 years of age) versus older YPLWH (over 18 years of age)?

2. If researchers want to enroll YPLWH in research, do you see any potential ethical or other problems with researchers being able to search medical records and clinical databases to find YPLWH that meet the criteria for a research study?

3. How and where do you think researchers should approach YPLWH for potential involvement in research? (E.g., in a clinic setting during routine care, in a community setting like a school, at home through a community health or outreach worker, etc.)

4. Especially for YPLWH who are younger (under 18 years of age), how should caregivers be involved in decisions related to the participation of YPLWH in research? Should caregivers be required to provide their consent for YPLWH to participate? What about for YPLWH who are over 18 years of age?

5. What are some of the risks of YPLWH participating in clinical research? In other words, do you think there are any negative consequences or bad things that could happen to YPLWH by participating in research?

Probe: Accidental issues of disclosure of HIV status, HIV-related stigma and discrimination

6. Do you think there are certain things that make YPLWH more vulnerable to risks or potential negative consequences compared to other groups of potential research participants? Are there things researchers should do to specifically protect YPLWH in research?

7. What are some of the benefits of YPLWH participating in clinical research? In other words, do you think there are positive things that could happen to YPWH by participating in research?

8. Some research involves collecting and analyzing participants’ blood. Do you have any concerns about research that collects and analyzes blood from participants? What are these concerns? What about for research with YPLWH, are there specific concerns or recommendations?

Probe: Responsibilities of researchers to provide feedback/share results of any tests

9. Some research involves the storage of participants blood, either to use later in the research study or to keep in a research laboratory to potentially use for research at a later time. Do you have any concerns about research that collects and then stores blood from participants? What are these concerns? What about for research with YPLWH, are there specific concerns or recommendations?

10. Some research, after they have stored blood from participants for a research project, want to do tests on that participant’s blood for reasons that are different from the original research study. Do you think researchers should be required to contact that participant and get permission to do additional tests on their blood? Do you have concerns about this and why? What about for research with YPLWH, are there specific concerns or recommendations?

11. Some research, after they have stored blood from participants for a research project, want to share blood samples with other researchers or organizations like governments who are interested in building up large databases of samples. Do you think researchers should be required to contact that participant and get permission to share their blood samples with other researchers or organizations? Do you have concerns about this and why? What about for research with YPLWH, are there specific concerns or recommendations?

12. In research that does store participants blood in a research lab, there are different ways to identify that blood sample. For example, sometimes that blood sample can be stored with participants’ name and other identifiable information, or it can be stored using a random study number so that we do not know the name of the person that the sample comes from. What do you think are the benefits and risks of storing participants specimens in these different ways? Would you recommend that researchers stored them in a specific way? What about for research with YPLWH, are there specific concerns or recommendations?

13. Are you involved in research with YPLWH? How so?

14. If you are involved in research with YPLWH, what are some of the ethical challenges you have faced in this research? How have you attempted to handle or address these challenges?

15. What guidelines and policies are in place in your setting to inform ethical research with YPLWH?

16. Are you involved in research with biological sampling and biobanking? How so?

17. If you are involved in research with biological sampling and biobanking, what are some of the ethical challenges you have faced in this research? How have you attempted to handle or address these challenges?

18. What guidelines and policies are in place in your setting to inform ethical research with biological sampling and biobanking?
